# Supplementary material for: POH1 deubiquitinates pro-interleukin-1β and restricts inflammasome activity
Source: Nat Commun. 2018 Oct 12;9:4225. doi: 10.1038/s41467-018-06455-z (PMC6185913; doi:10.1038/s41467-018-06455-z)
Supplement: Supplementary file 1 — Supplementary Information [file 41467_2018_6455_MOESM1_ESM.pdf]

Supplementary Information

POH1 deubiquitinates pro-interleukin-1β and restricts inflammasome activity

Zhang *et. al.*

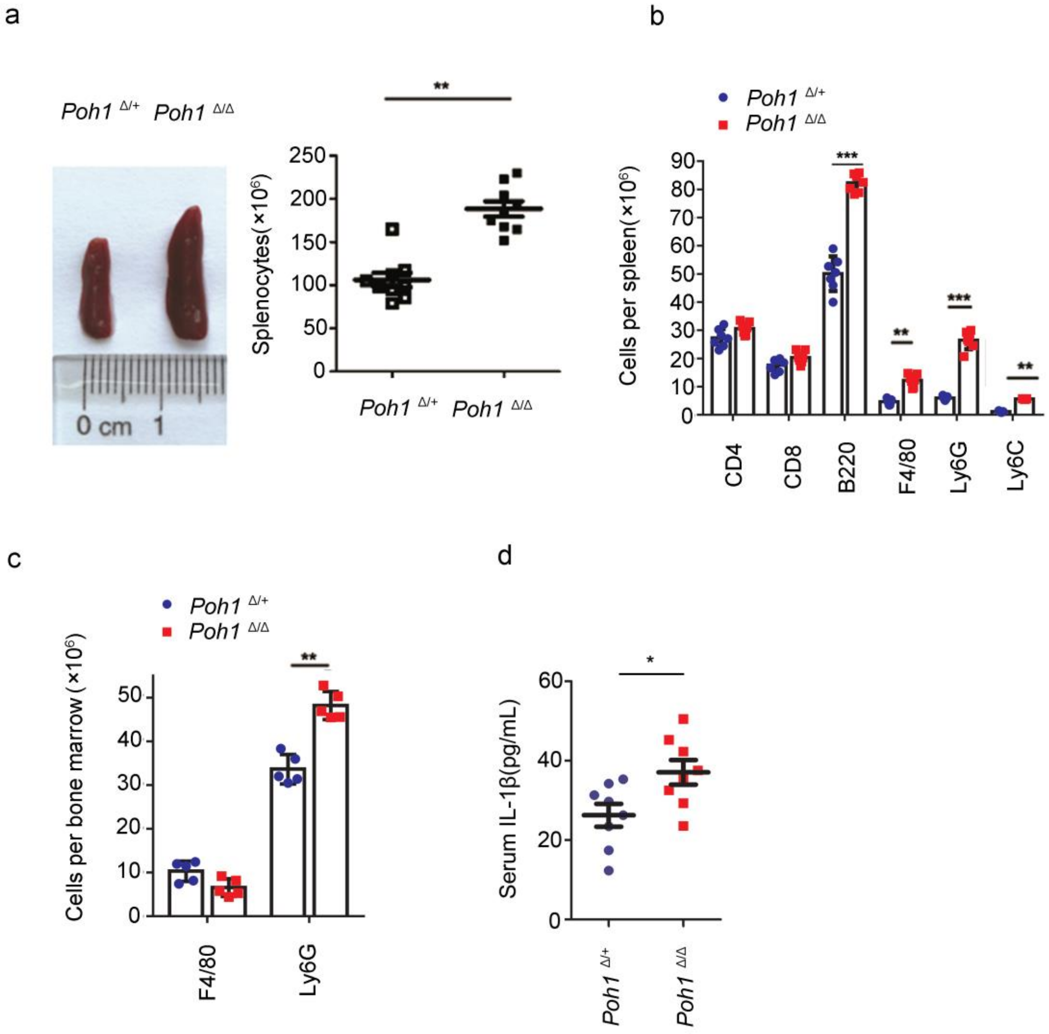

S1

Supplementary Figure 1. a Morphology (left panel) and cell numbers (right panel) of spleens in *Poh1*$^{\Delta/+}$ and *Poh1*$^{\Delta/\Delta}$ mice (n=9 per group). **b, c** Single cell suspensions were prepared from the spleen and bone marrow, (**b**) absolute numbers of CD4^{+} or CD8^{+} T cells, B220^{+} B cells, CD11b^{+} F4/80^{+} macrophages, CD11b^{+} Ly6G^{+} neutrophils and CD11b^{+} Ly6C^{+} monocytes in the spleens of *Poh1*$^{\Delta/+}$ and *Poh1*$^{\Delta/\Delta}$ mice were measured (n=7 per group); or (**c**) absolute numbers of CD11b^{+} F4/80^{+} macrophages and CD11b^{+} Ly6G^{+} neutrophils in the bone marrow (n=5 per group) were measured. **d** ELISA quantification of the IL-1β levels in the serum of *Poh1*$^{\Delta/+}$ and *Poh1*$^{\Delta/\Delta}$ mice

(n=8 mice per group). Data are pooled from **(a)** three or **(b-d)** two independent experiments (mean ± s.d. in **a-c**; mean ± s.e.m. in **d**), *p<0.05, **p<0.01, *** p<0.001 (two tailed Student's t-test).

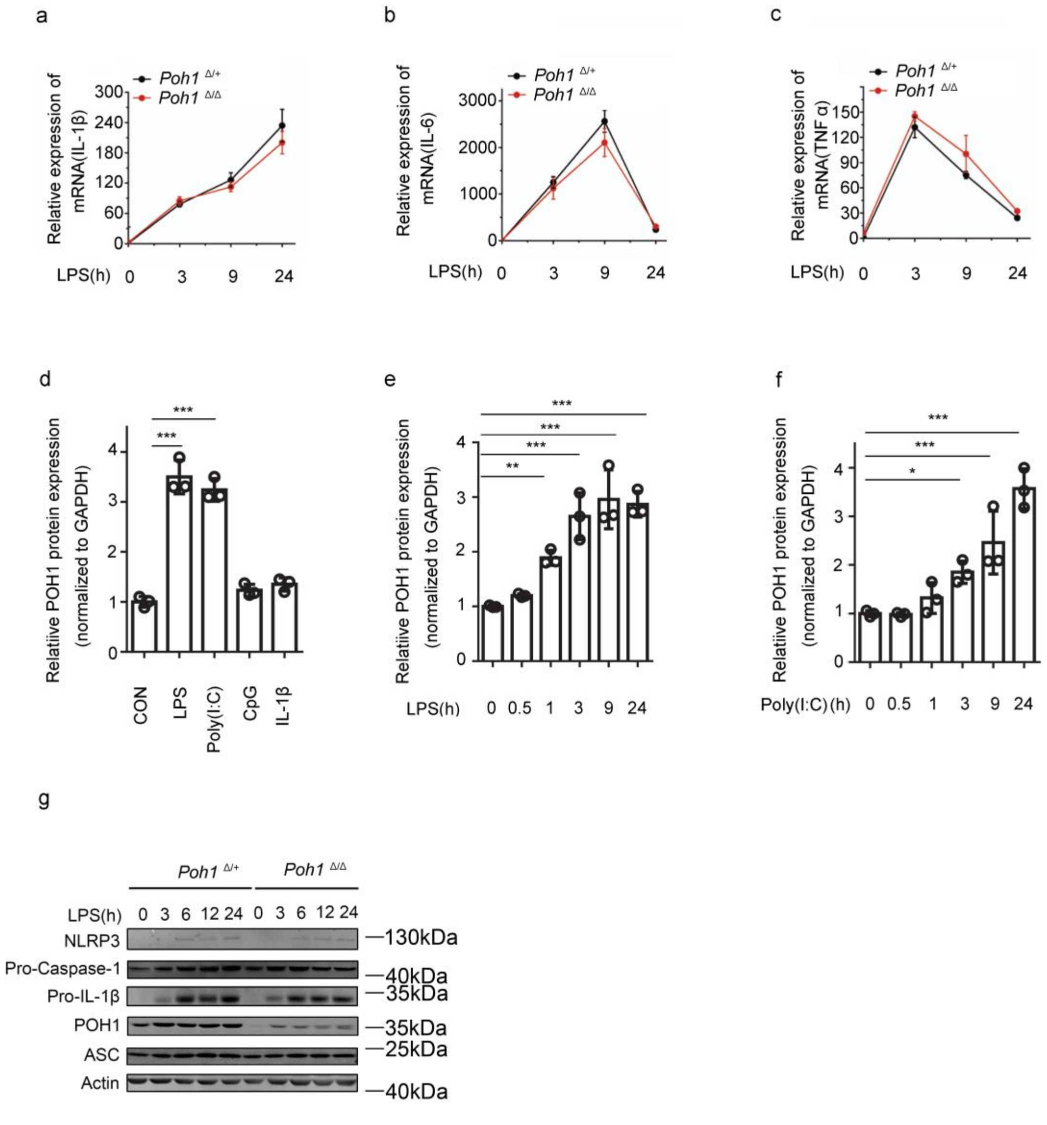

S2

Supplementary Figure 2. a-c BMDMs from *Poh1*$^{\Delta/+}$ and *Poh1*$^{\Delta/\Delta}$ mice were treated with LPS as indicated, then the mRNA levels of (**a**) IL-1β, (**b**) IL-6 and (**c**) TNFα were measured by RT-PCR. Similar results were obtained from three independent experiments. The results represent the

mean ±s.d. of three independent sets of experiments. **d-f** Quantification of western blot signal intensity for **(d)** Fig. 2a, **(e)** Fig. 2b and **(f)** Fig. 2c from three independent experiments, data presented as the mean ±s.d. , *p<0.05, **p<0.01, *** p<0.001 (one-way ANOVA with Dunnett's post-hoc test). **g** BMDMs from *Poh1*$^{\Delta/+}$ and *Poh1*$^{\Delta/\Delta}$ mice were treated with LPS as indicated, then the cell lysates were IB with the indicated antibodies. Similar results were obtained from three independent experiments.

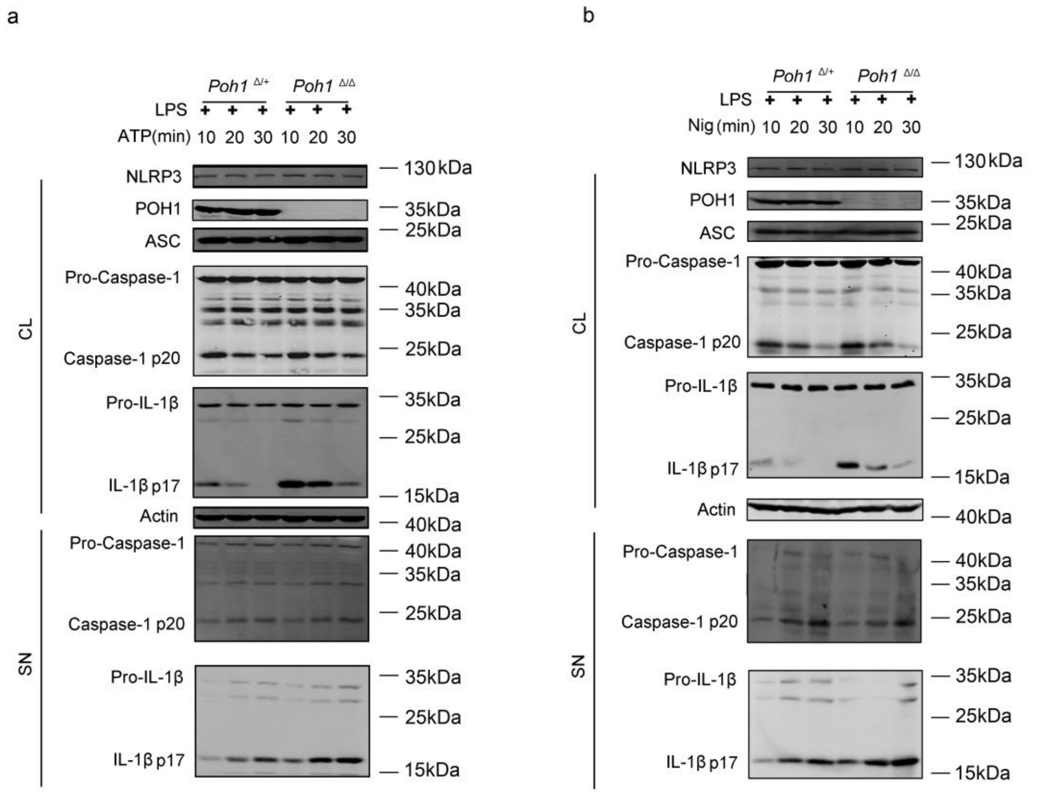

S3

Supplementary Figure 3. a, b BMDMs from *Poh1*$^{\Delta/+}$ and *Poh1*$^{\Delta/\Delta}$ mice were primed with LPS, then treated with **(a)** ATP and **(b)** nigericin as indicated. The cell lysates and supernatants were IB with the indicated antibodies. Similar results were obtained from three independent experiments.

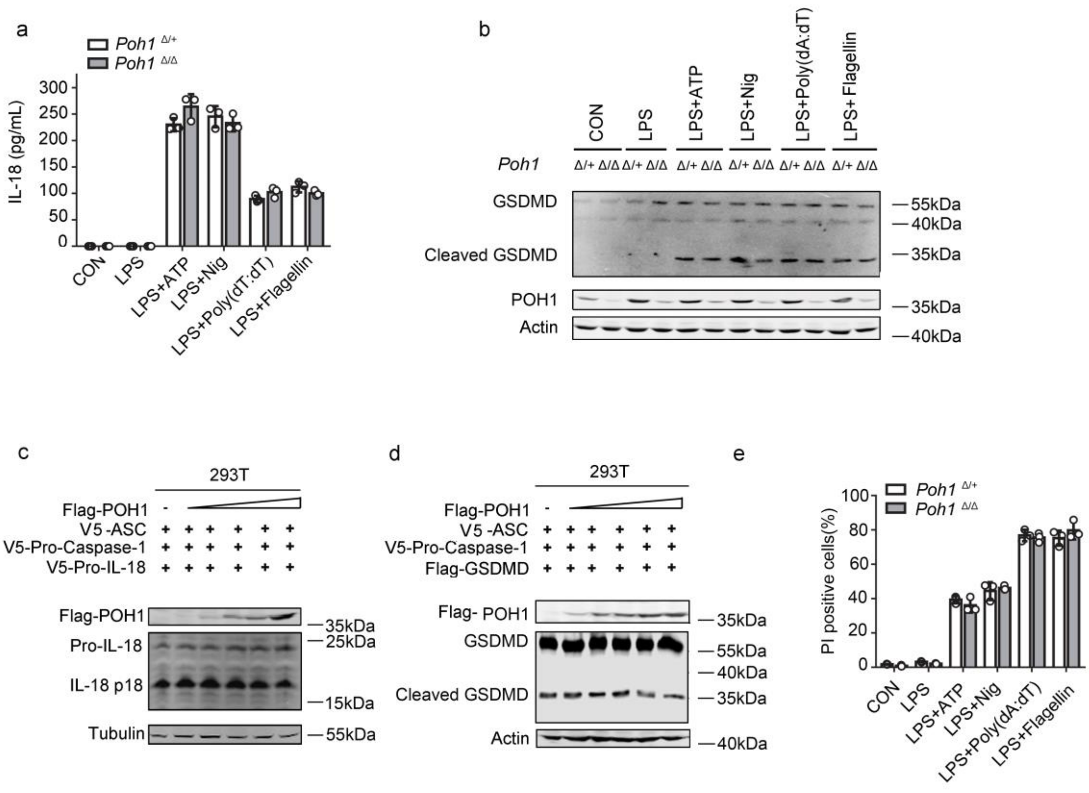

S4

Supplementary Figure 4. a, b *Poh1*$^{\Delta/+}$ and *Poh1*$^{\Delta/\Delta}$ BMDMs were primed with LPS for 12-14 hrs and then stimulated with inflmmasome agonists as indicated. **(a)** The IL-18 levels in supernatants were measured by ELISA. **(b)** The cell lysates were IB with the indicated antibodies. **c, d** HEK293T cells were transfected with Flag-POH1, V5-caspase-1, V5-ASC and along with **(c)** V5-pro-IL18 or **(d)** Flag-GSDMD, then the cell lysates were subjected to IB with the indicated antibodies. **e** BMDMS were treated as in **a**, then cell pyroptosis was measured by PI staining. Similar results were obtained from three independent experiments. The results represent the mean ± s.d. of three independent sets of experiments.

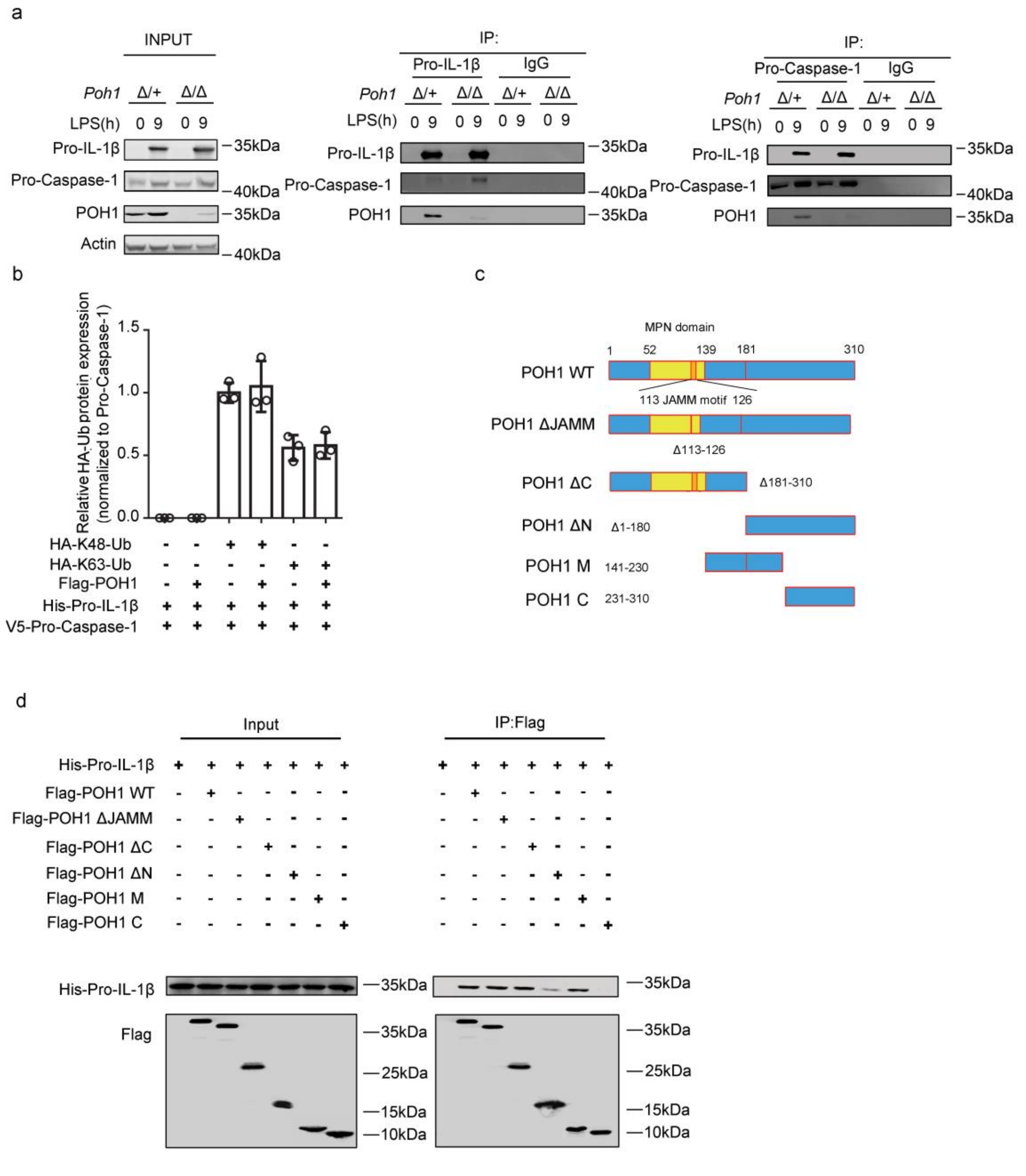

S5

Supplementary Figure 5. a BMDMs from *Poh1*$^{\Delta/+}$ and *Poh1*$^{\Delta/\Delta}$ mice were stimulated with LPS for 9 hrs, then cells were subjected to IP with an anti-pro-IL-1β or an anti-pro-caspase-1 antibody and IB with the indicated antibodies. **b** Quantification of ubiquitinated pro-caspase-1 for Fig. 5d from three independent experiments, data presented as the mean ± s.d. **c** Schematic diagram of mutants of POH1. **d** HEK293T cells transfected with a plasmid encoding His-pro-IL-1β, Flag-POH1 (WT) or its mutants were subjected to IP with an anti-Flag antibody and IB with the indicated antibodies.

Similar results were obtained from three independent experiments.

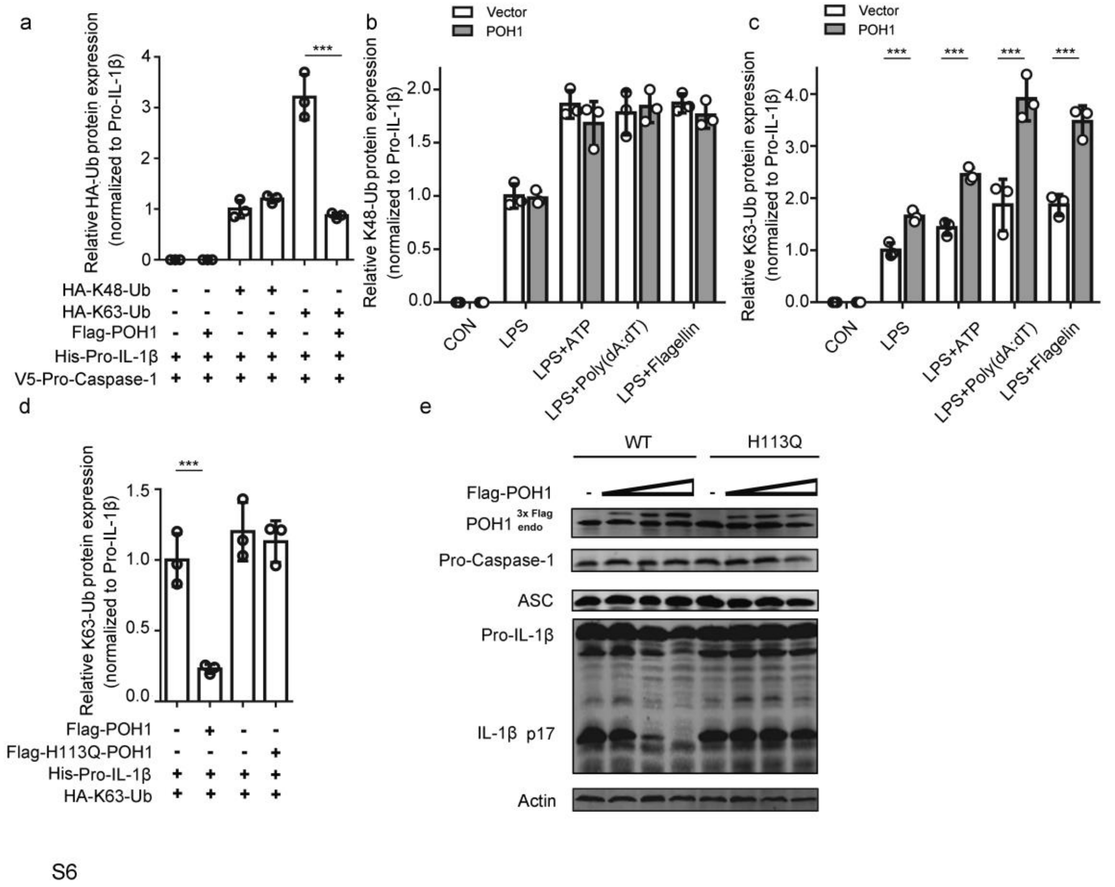

Supplementary Figure 6. a-d Quantification of ubiquitinated pro-IL-1β for **(a)** Fig. 5d, **(b-c)** Fig. 5e and **(d)** Fig. 5f from three independent experiments, data presented as the mean ±s.d., *** p<0.001. (two tailed Student's t-test). **e** HEK293T cells were transfected with Flag-caspase-1, Vsv-ASC, His-pro-IL-1β, Flag-POH1 (WT) or Flag-H113Q-POH1, then cell lysates were subjected to IB with the indicated antibodies. Similar results were obtained from three independent experiments.

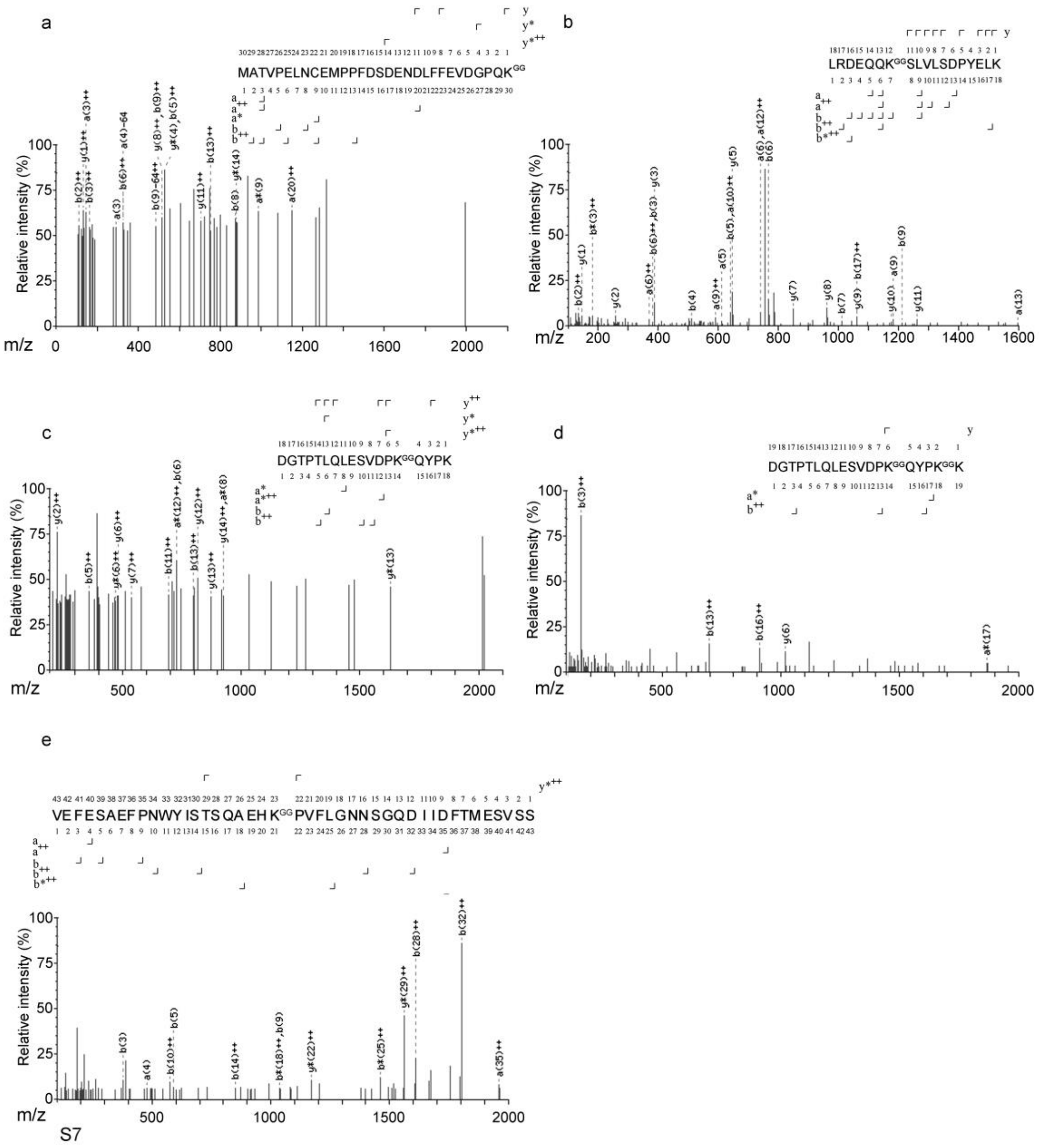

Supplementary Figure 7. a-e HEK293T cells transfected with a plasmid encoding His-pro-IL-1β, Flag-ASC, V5-pro-caspase-1 and HA-K63-only Ub were subjected to IP with an anti-His antibody and prepared for MS analysis. Sequence ions are labelled (C-terminal ione, up the sequence of indicated peptide; N-terminal ions, below the sequence of indicated peptide). Data-base search identifies 5 peptides of pro-IL-1β, containing GG (diglycine) remnant on (**a**) K30 (theoretical monoisotopic m/z = 3616.5320), **(b)** K133 (theoretical monoisotopic m/z =2274.1855), **(c)** K205 (theoretical monoisotopic m/z = 2057.0317), **(d)** K205/K209 (theoretical monoisotopic m/z =

2413.2125) and **(e)** K247 (theoretical monoisotopic m/z = 4836.2177) residues.

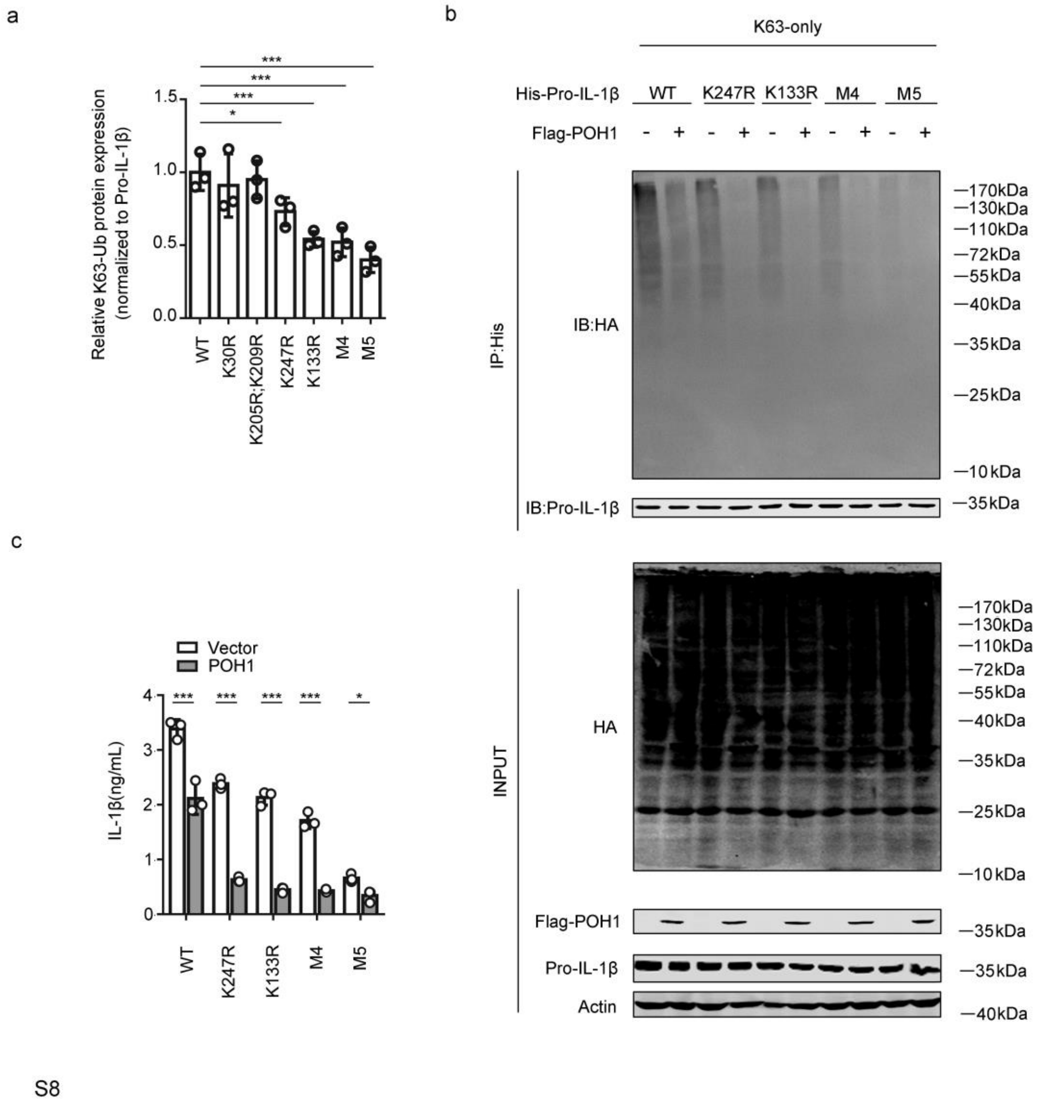

Supplementary Figure 8. a Quantification of ubiquitinated pro-IL-1β for Fig. 6d from three independent experiments, data represent the mean ± s.d., **p*<0.05, *** *p*<0.001 (one-way ANOVA with Dunnett's post-hoc test). **b, c** HEK293T cells were transfected with Flag-POH1, V5-caspase-1, V5-ASC, His-pro-IL-1β (WT) or its mutants, along **(b)** with or **(c)** without HA-tagged K63-only

Ub, then (**b**) cells were subjected to IP with an anti-His antibody and IB with the indicated antibodies; (**c**) The IL-1β levels in supernatants were measured by ELISA. Similar results were obtained from three independent experiments. The results represent the mean ± s.d. of three independent sets of experiments. * $p<0.05$, *** $p<0.001$ (two tailed Student's t-test).

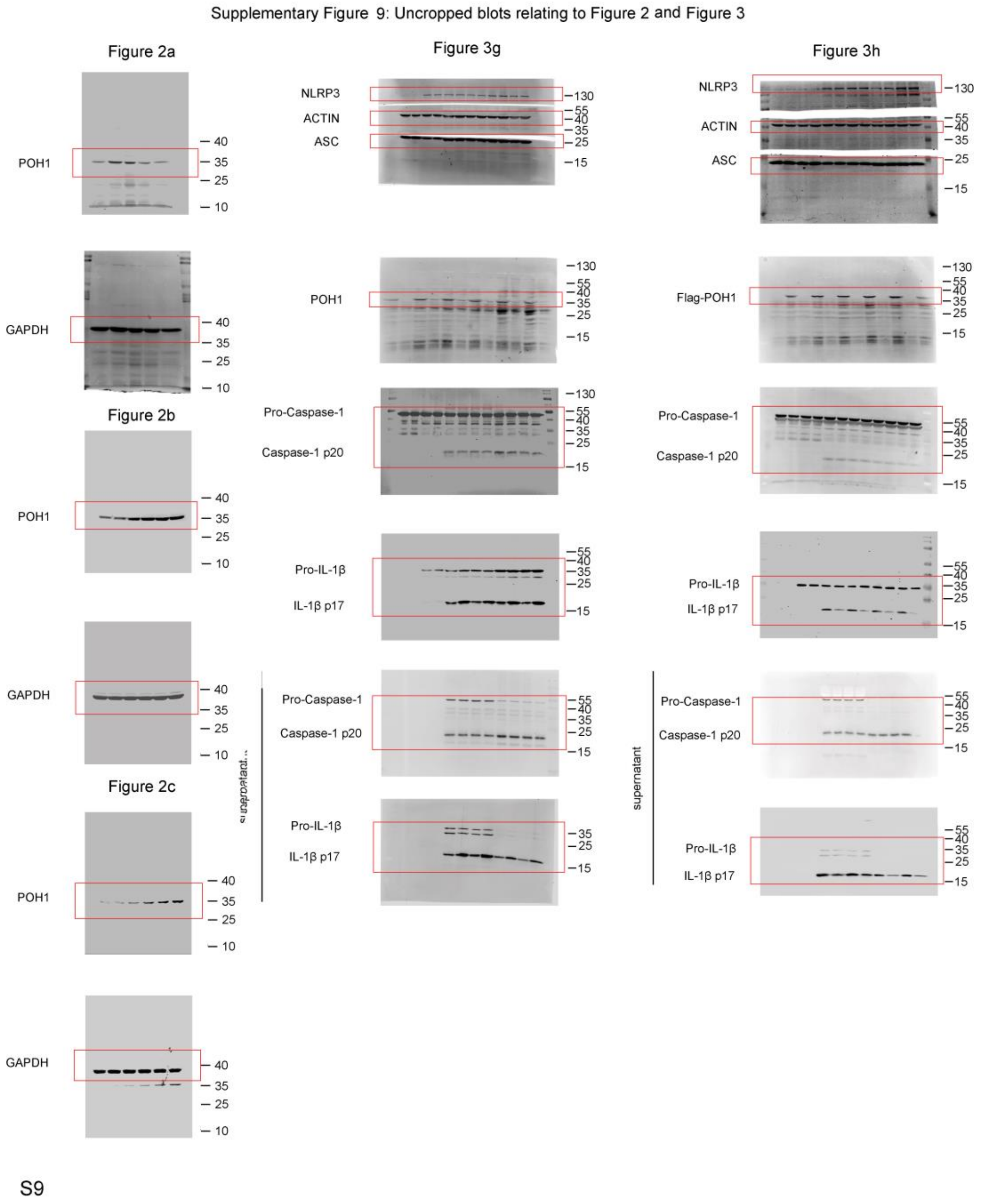

Supplementary Figure 9. Uncropped blots relating to Figure 2 and Figure 3.

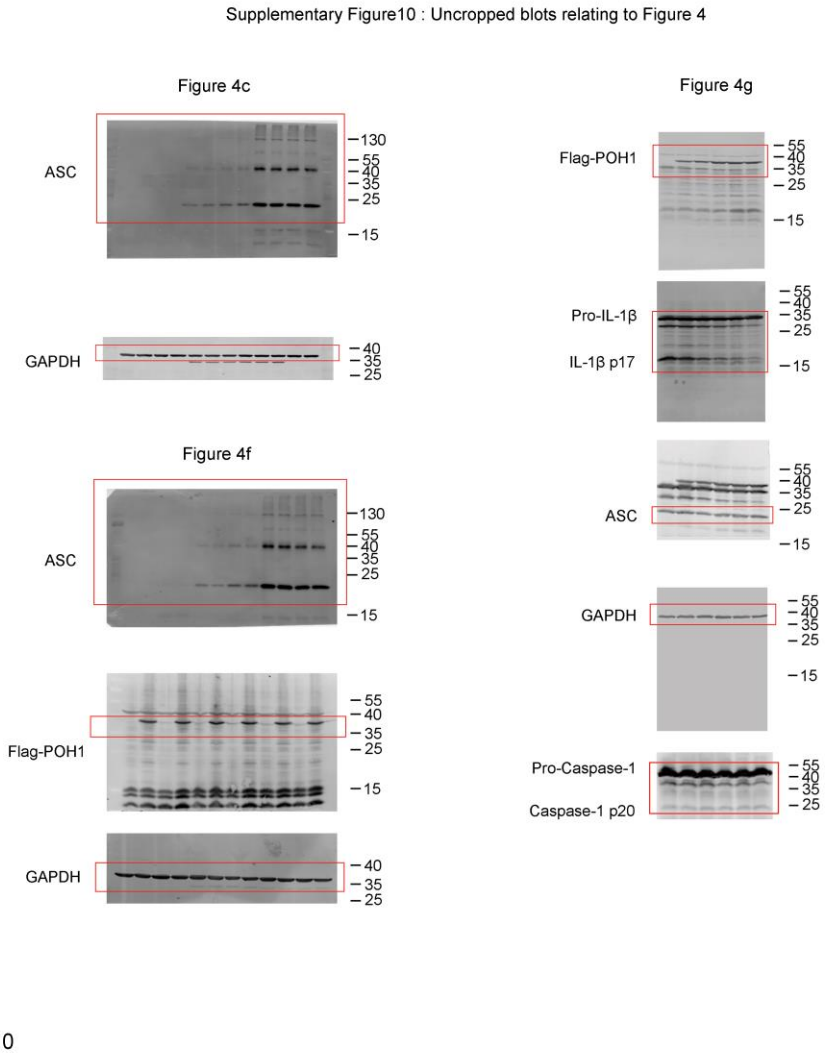

Supplementary Figure 10. Uncropped blots relating to Figure 4.

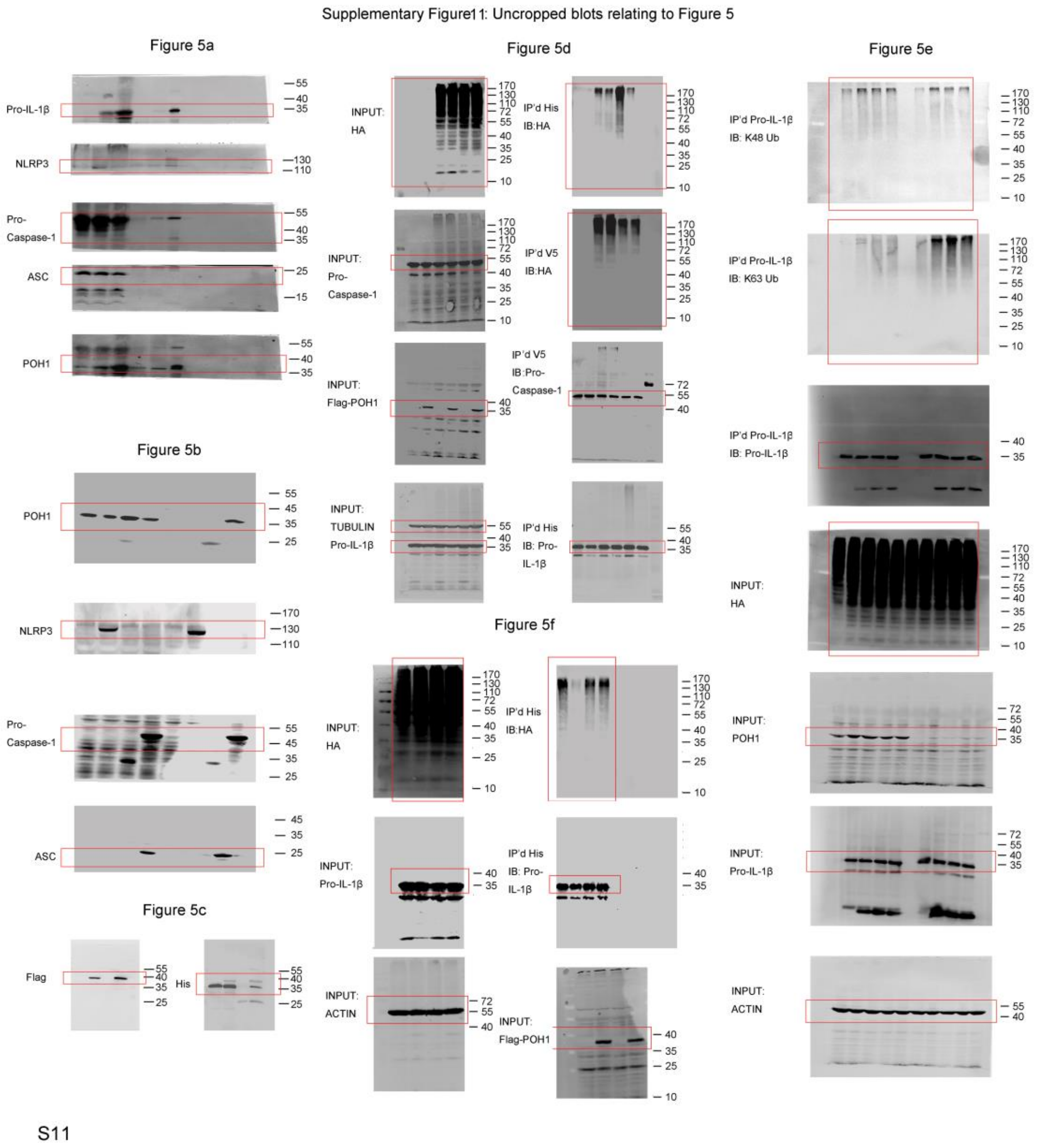

Supplementary Figure 11. Uncropped blots relating to Figure 5.

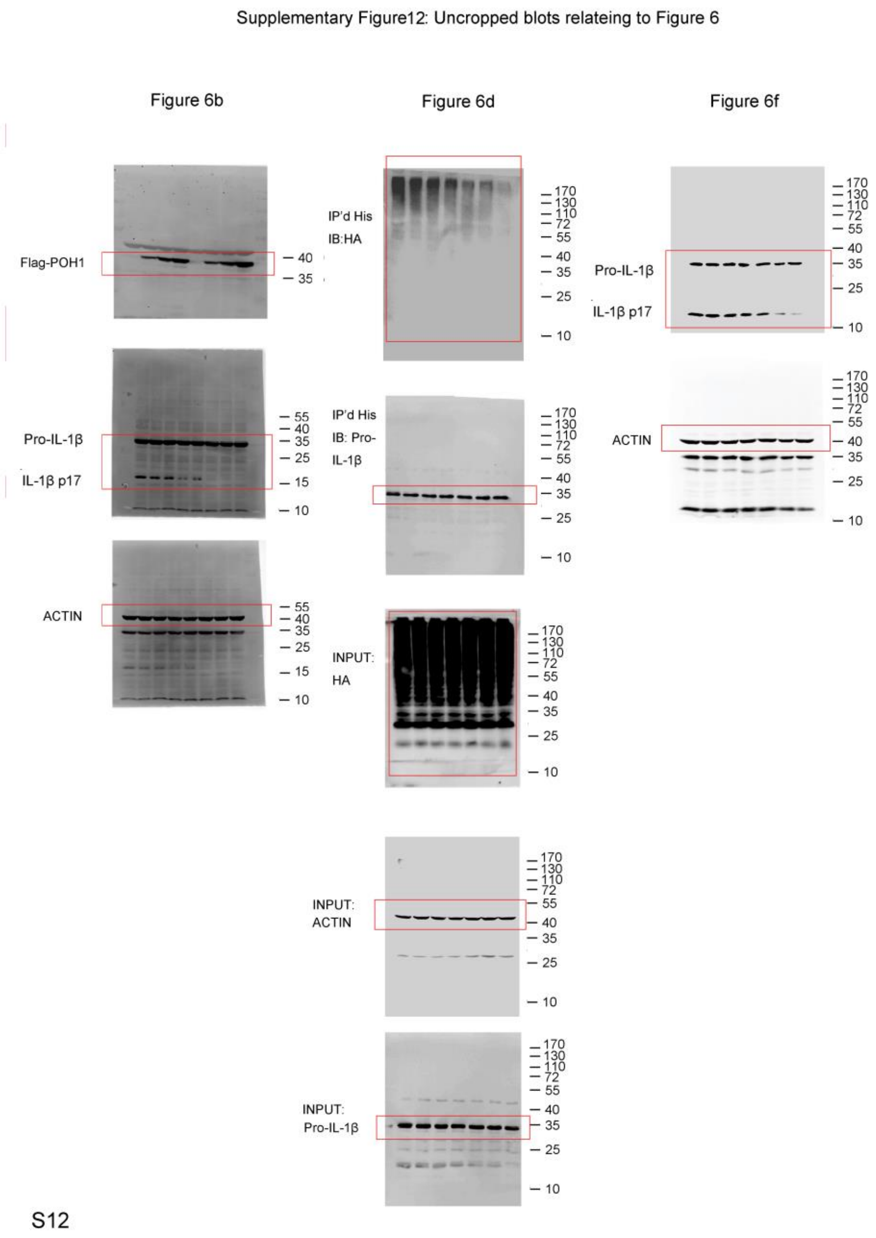

Supplementary Figure 12. Uncropped blots relating to Figure 6.

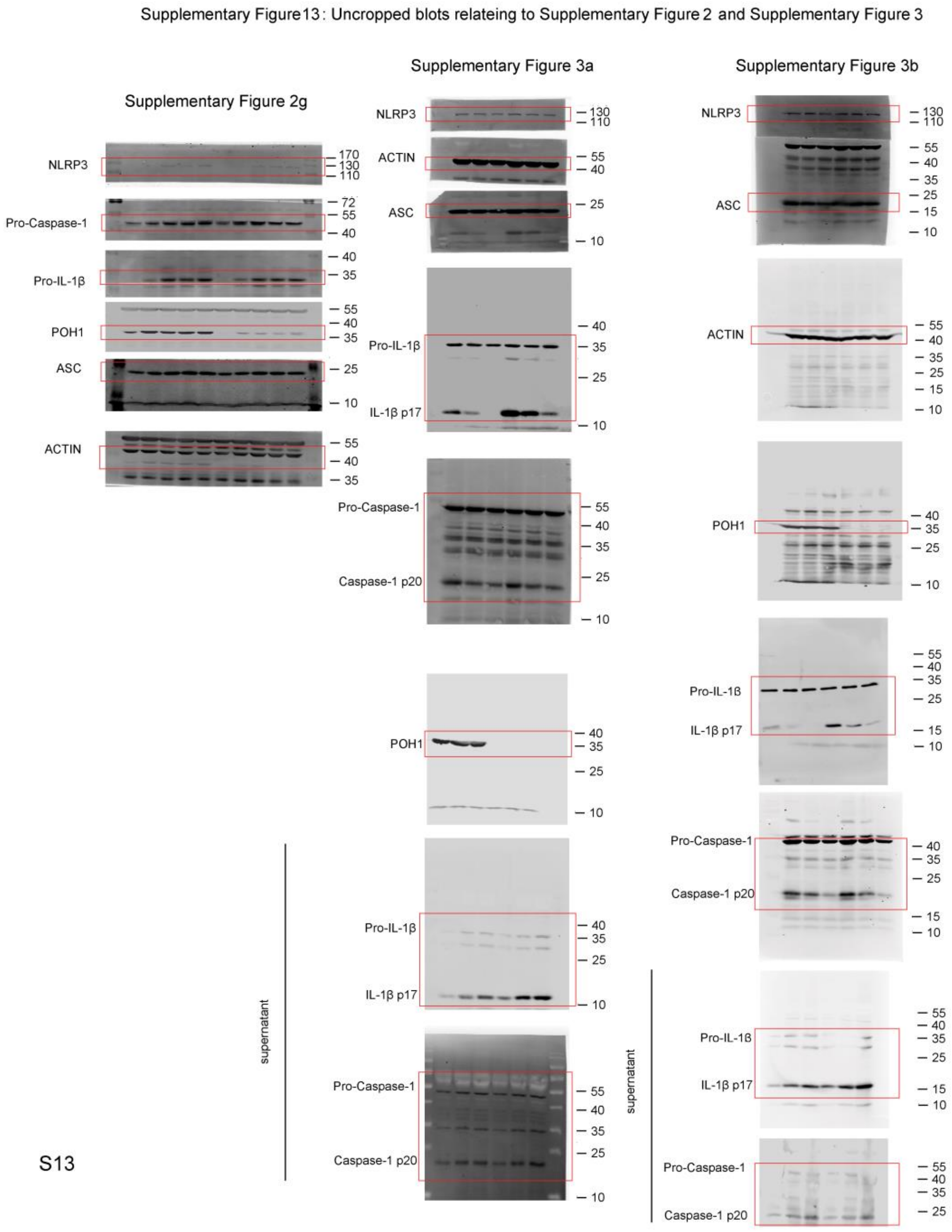

Supplementary Figure 13. Uncropped blots relating to Supplementary Figure 2 and Supplementary Figure 3.

Supplementary Figure14: Uncropped blots relateing to Supplementary Figure 4

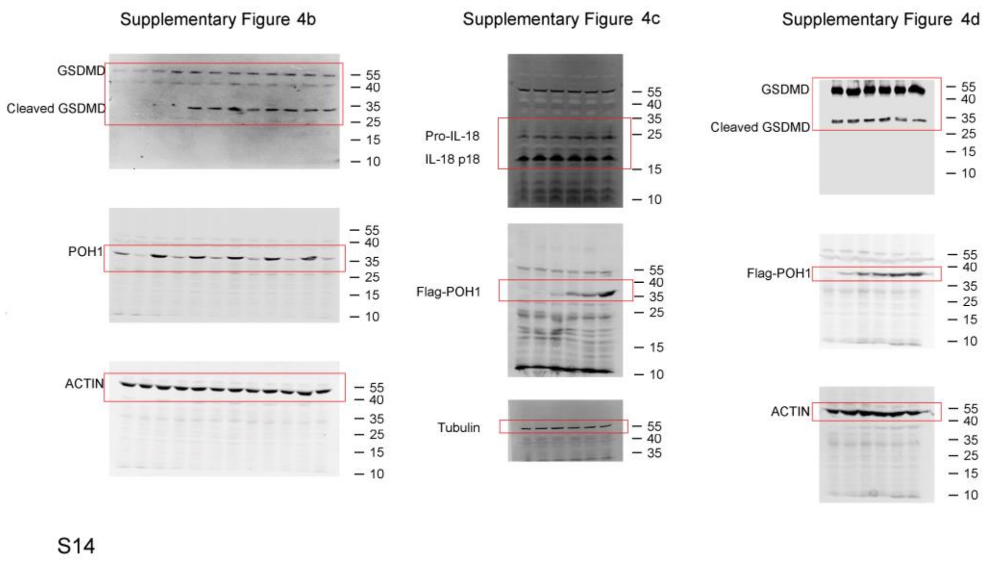

Supplementary Figure 14. Uncropped blots relating to Supplementary Figure 4.

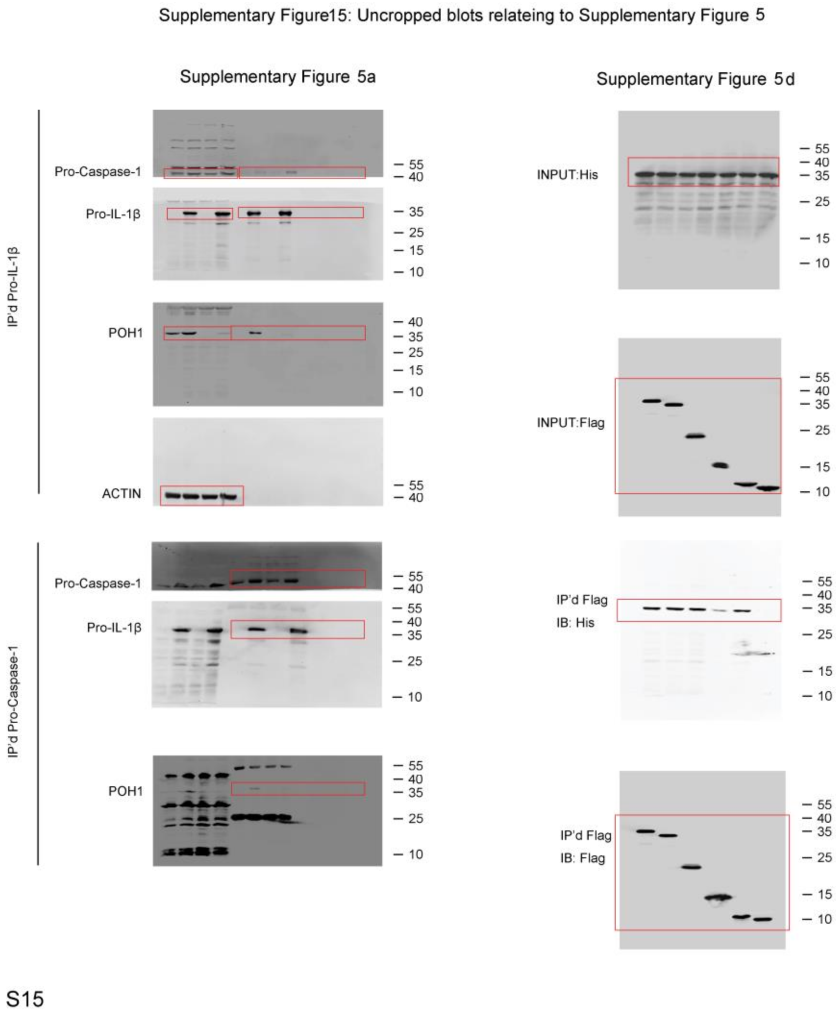

Supplementary Figure 15. Uncropped blots relating to Supplementary Figure 5.

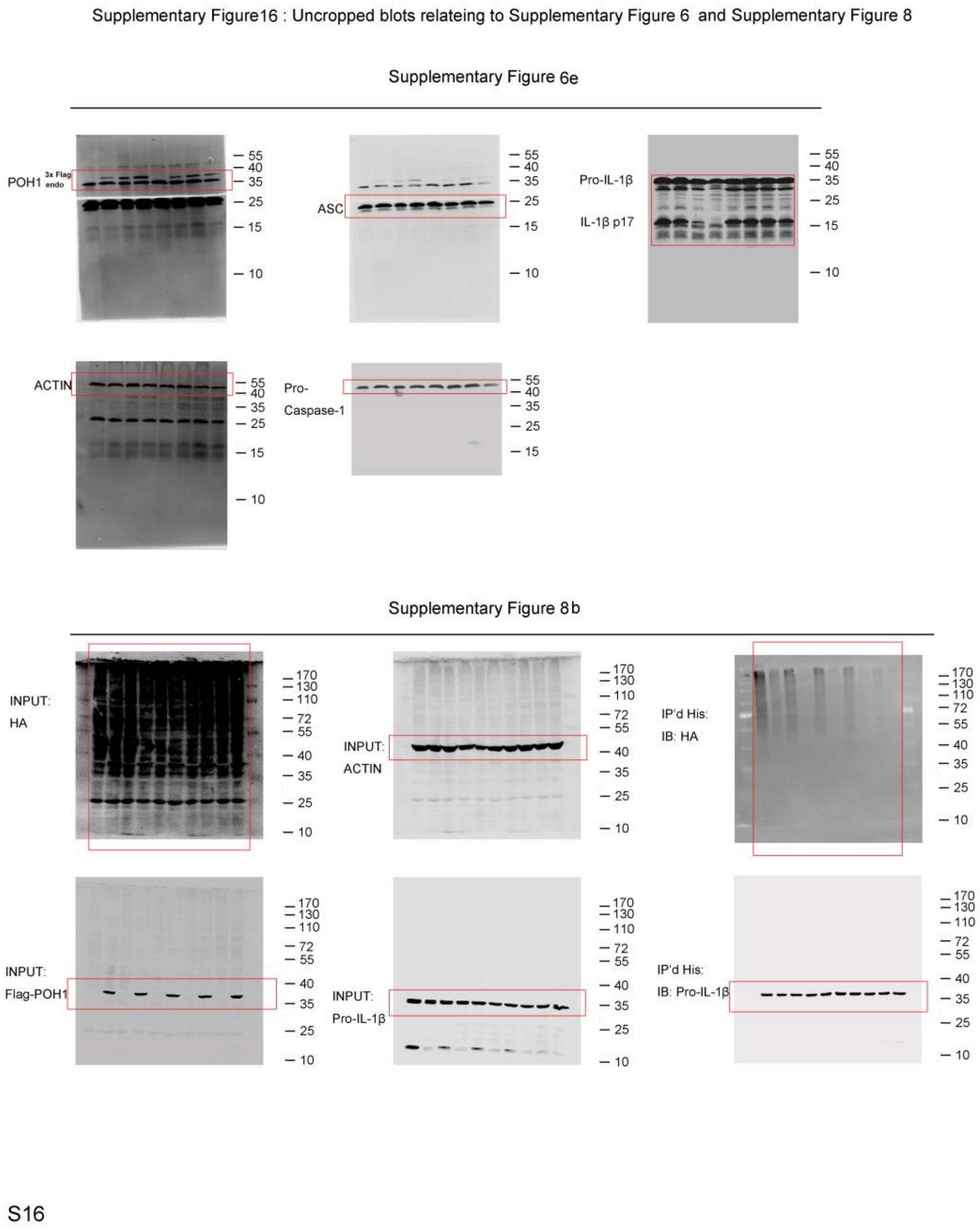

Supplementary Figure 16. Uncropped blots relating to Supplementary Figure 6 and Supplementary Figure 8.

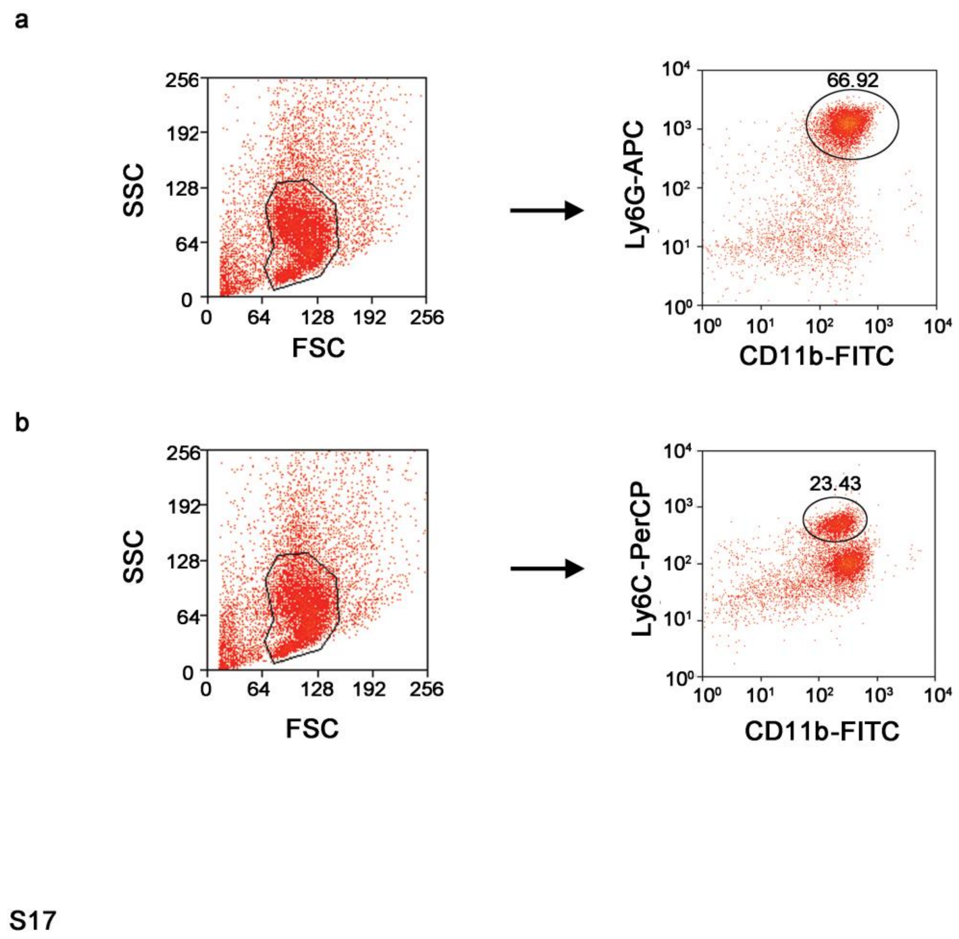

Supplementary Figure 17. a, b Example flow spectrometry gating strategy for peritoneal **(a)** neutrophils and **(b)** monocytes.

Supplementary Table 1. Primers used in this study for Real-Time PCR

**Primers**	**Sequences**
Mus *GAPDH* forward	5'-AGGTCGGTGTGAACGGATTTG-3'
Mus *GAPDH* reverse	5'-TGTAGACCATGTAGTTGAGGTCA-3'
Mus *IL-1β* forward	5'-TCCAGGATGAGGACATGA-3'
Mus *IL-1β* reverse	5'-GAACGTCACACACCAGCAGGTTA-3'
Mus *IL*-*6* forward	5'-ACAAAGAAATGATGGATGCTACC-3'
Mus *IL*-*6* reverse	5'-GTTGTTCTTCATGTACTCCAGGT-3'
Mus *TNFα* forward	5'-AAGCCTGTAGCCCACGTCGTA-3'
Mus *TNFα* reverse	5'-GGCACCACTAGTTGGTTGTCTTTG-3'
Mus *POH1* forward	5'-AGTGCCTATGGAAGTTATGGGT-3'
Mus *POH1* reverse	5'-CTGCTTCAACACTGACACCA-3'
